# Cadmium Toxicity in *Caenorhabditis elegans*: Mechanisms and Interventions by Vitamin C and Fruit Juices

**DOI:** 10.3390/foods14173106

**Published:** 2025-09-05

**Authors:** Yanyan Zhao, Shan Wang, Hongyan Zhang, Xingru Lu, Hengxi Sun, Huiling Hu, Liangbin Hu, Ligong Zhai, Wei Zhou, Haizhen Mo

**Affiliations:** 1 School of Food Science and Engineering, Anhui Science and Technology University, Wenhui Road, Chuzhou 239099, China; zhaoyanyan@ahstu.edu.cn (Y.Z.);; 2 School of Food Science and Technology, Henan Institute of Science and Technology, No. 655 Hualan Street, Xinxiang 453003, China; 3 School of Horticulture and Landscape Architecture, Henan Institute of Science and Technology, Xinxiang 453003, China; hu-huiling@163.com; 4 Department of Food and Bioengineering, Shaanxi University of Science and Technology, Weiyang Lake Street, Xi’an 710021, China; 5 Anhui Province Key Laboratory of Functional Agriculture and Functional Food, Anhui Science and Technology University, Chuzhou 239000, China

**Keywords:** cadmium, *Caenorhabditis elegans*, ascorbic acid, reactive oxygen species, nitric oxide

## Abstract

With the rapid development of industry, heavy metal pollution has emerged as a significant threat to food safety and human health. Among these pollutions, cadmium (Cd) pollution has become a global environmental concern. *Caenorhabditis elegans*, with its short life cycle and evolutionary conservation with humans, serves as an ideal model organism for studying toxicity mechanisms. In this study, we investigated the mechanisms of toxicity induced by Cd in *C. elegans* and the intervention of ascorbic acid (V_C_) and fruit juices on toxicity induced by Cd. Using fluorescent probes, we observed that Cd exposure (5 mg/mL and 8 mg/mL of CdCl_2_) significantly decreased the vitality of *C. elegans* in a dose-dependent manner within 6 h. Reactive oxygen species (ROS) and nitric oxide (NO) accumulated concurrently. Further exploration revealed that ROS played a crucial role in Cd-induced acute toxicity. The inhibition of ROS by Imidazole (IMZ) and Pyridine (PY) not only reduced NO levels but also restored the vitality of *C. elegans*. Conversely, the removal of NO by cPTIO [2-(4-Carboxyphenyl)-4,4,5,5-tetramethylimidazoline-1-oxyl-3-oxide] or L-NMMA (N^G^-Monomethyl-L-arginine) improved the vitality; however, it still did not affect ROS levels, indicating that ROS accumulation is a primary event in Cd-induced toxicity. We also examined the protective effects of V_C_ and fruit juices. Both V_C_ (0.5 mg/mL, 1 mg/mL) and fruit juices (50% (*v*/*v*) of the total volume of the medium) significantly enhanced the vitality of *C. elegans* under CdCl_2_ stress and reduced the accumulation of ROS and NO. This suggests that V_C_-rich materials effectively scavenge ROS and NO, thereby alleviating Cd-induced oxidative stress and toxicity. In summary, our results elucidate that CdCl_2_ exposure leads to ROS accumulation in *C. elegans*, which in turn triggers NO production and ultimately reduces nematode activity. V_C_ and V_C_-rich materials can effectively mitigate CdCl_2_ toxicity by scavenging ROS and NO, providing a potential strategy for alleviating Cd poisoning.

## 1. Introduction

With the rapid advancement of industrialization, heavy metal pollution of agricultural land has become an increasingly severe issue, posing a significant threat to food safety and human health. Among these heavy metals, Cd has garnered substantial attention due to its high toxicity, persistence in the environment, and bioaccumulation potential [1]. Cd constitutes a significant risk to human health, inducing both acute and chronic adverse effects on various organs and physiological systems [2]. Cd is introduced into the body through multiple pathways, including bioaccumulation via the food chain, inhalation from environmental sources such as smoking, and dermal contact [3]. Cd is a non-biodegradable heavy metal that can cause toxicity at low concentrations [4]. In addition, it is hard to remove Cd permanently from the environment [5]. As a result, addressing Cd pollution is of urgent importance to protect public health and the environment.

The bioactive compounds in food exhibit a wide range of biological activities, such as anticancer, antioxidant, immune regulation, antimicrobial, cholesterol-lowering, antiaging, and other activities [6]. Thus, bioactive compounds, predominantly found in foods, may serve as a viable therapeutic approach for mitigating Cd-induced renal damage [4]. The potential of these bioactive compounds to ameliorate acute cadmium poisoning warrants further investigation and may represent a valuable approach in the management of cadmium toxicity. V_C_, as a bioactive compound in food, may be the most important water-soluble antioxidant in human plasma. V_C_ plays an essential role in immune function, collagen, and cortisol synthesis, and it could remove free radical intermediates that initiate damaging reactions [7,8]. Fruit juices contain a variety of bioactive components, such as V_C_, polysaccharides, and polyphenolic compounds, all of which offer numerous health benefits, including antioxidant properties. Given its antioxidant properties, V_C_ and V_C_-rich fruit juices may effectively reduce Cd toxicity by scavenging ROS.

Citrus fruits are abundant reservoirs of phytochemicals, including flavonoid glycosides, coumarins, volatile oils, β- and γ-sitosterol, and the rare polyethoxylated flavones. Their dietary matrix is further enriched with polyphenolic compounds, notably ascorbic acid, which effectively prevents V_C_ deficiency [9]. Oranges and lemons both belong to the citrus family. Owing to their elevated Vc content, orange and lemon juices were used as test matrices to quantify their potential protective efficacy against Cd-mediated toxicity in *C. elegans*.

With a short life cycle, exceptional genetic tractability, and profound evolutionary conservation, *C. elegans* is an outstanding model for studying toxicity mechanisms [10,11]. This small nematode recapitulates the major organ systems of higher vertebrates, such as nervous, digestive, muscular, hypodermal, and reproductive systems. It displays extensive genetic homology to humans and exhibits sensitivity to environmental toxicants [12]. And its numerous genes and pathways are similar to those responsible for human diseases [13]. These features make *C. elegans* a valuable tool for studying the toxic effects of Cd and evaluating potential interventions.

The development, reproduction, intestinal ROS production, and locomotion behavior of *C. elegans* can be used to assess the toxic effects of specific toxicants or stresses [14,15]. Moreover, *C. elegans* shows the potential for evaluating the ecological risk of specific toxicants at environmentally relevant concentrations [16].

In this study, we aimed to investigate the mechanisms underlying Cd toxicity in *C. elegans* and explore the potential protective effects of V_C_ and fruit juices. Our findings may provide insights into the role of V_C_ in mitigating heavy metal toxicity and offer a possible strategy for alleviating Cd poisoning.

## 2. Materials and Methods

### 2.1. Strains and Culture Conditions

Food medium: Briefly, *Escherichia coli* OP50 (Caenorhabditis Genetics Center, Minneapolis, MN, USA) stocks were inoculated in liquid broth medium (LB: 10 g/L of NaCl, 10 g/L of peptone, 5 g/L of yeast extract) at 37 °C for 16 h. The bacteria were collected by centrifugation and resuspended in LB medium. Then, 2 mL of the mixture was spread onto the nematode growth medium agar plates. The plates were then exposed to ultraviolet light for 30 min to kill the *E. coli* OP50. After the plates were dried, they were collected for subsequent use.

The nematode growth medium (NGM: 3 g/L of NaCl, 2.5 g/L of peptone, 17 g/L of agar, 25 mL of KH_2_PO_4_-K_2_HPO_4_ buffer, 100 μL of 1 mol/L of CaCl_2_, 1 mL of 5 mg/mL cholesterol, 100 μL of 1 mol/L MgSO_4_) was prepared.

*C. elegans* (Caenorhabditis Genetics Center, Minneapolis, MN, USA) were cultured at 20 °C and 70% relative humidity in darkness on NGM agar seeded with *E. coli* OP50.

### 2.2. C. elegans Synchronous Cultivation

*C. elegans* in the adult stage were washed with M9 buffer (2 mM MgSO_4_, 0.4% glucose, 0.1 mM CaCl_2_, 1/5 volume of 5*M9 (5*M9 buffer: 17.1 g/L Na_2_HPO_4_·12H_2_O, 3.0 g/L K_2_H_2_PO_4_, 0.5 g/L NaCl, 1.0 g/L NH_4_Cl)) two to three times. Then, 0.5 M NaOH and 0.5% NaClO were added, and the mixture was shaken slightly. The eggs of *C. elegans* were washed with M9 buffer more than three times.

### 2.3. Fruit Juice Preparation

Oranges and lemons were obtained from local markets in China. The oranges and lemons were peeled, and the edible parts were cut into small pieces and homogenized with Ultra-turrax T25 basic (IKA Co., Ltd., Staufen, Germany). The suspension was centrifuged at 8000× *g* at 4 °C for 5 min. Finally, the supernatant was filtered with a 0.22 μm sterile membrane. Determination of V_C_ was performed by DCPIP (2,6-dichloroindophenol) according to the Association of Official Analytical Chemists (AOAC) method.

### 2.4. Detection of the Vitality of C. elegans

To study the vitality of *C. elegans*, SYTOX Green, a nucleic acid-binding fluorescent dye, was used. SYTOX Green was excluded from live cells and entered the permeabilized cells [17]. *C. elegans* that had grown to the L4 stage were transferred to NGM containing *E. coli* OP50 and different concentrations of CdCl_2_ (0, 1, 5, and 8 mg/mL), and then incubated at 20 °C. *C. elegans* were collected at 0, 0.5, 1, 2, 4, and 6 h, and washed with M9 buffer, respectively. Then, SYTOX Green was added with a final concentration of 1 μM, and the mixtures were incubated at 20 °C for 15 min in the dark. Finally, the nematodes were washed with M9 buffer, and the fluorescence of SYTOX Green was quantified using a microreader with the excitation wavelength 488 nm and emission wavelength 525 nm (Thermol (Waltham, MA, USA), Varioskan Flash).

### 2.5. Analysis of ROS

DCFH-DA (dichlorodihydrofluorescein diacetate) was used to detect the level of ROS [18]. DCFH-DA (Beyotime Biotechnology Institute, Haimen, China) staining solution was added to the *C. elegans* at the L4 stage, and the ultimate concentration of the fluorescent probe was 1 μM. Then, different concentrations of CdCl_2_ (0, 5, and 8 mg/mL) were added to the mixtures, and the mixtures were incubated at 20 °C. The fluorescent intensity of *C. elegans* was analyzed at 2 h and 6 h using an excitation wavelength of 488 nm and an emission wavelength of 525 nm.

### 2.6. Analysis of NO

DAF-FM DA (4-amino-5-methylamino-2′,7′-difluorescein diacetate) was used to determine the level of NO [19]. When at the L4 stage, *C. elegans* were mixed with DAF-FM-DA (Beyotime Biotechnology Institute, Haimen, China), and the final concentration of DAF-FM-DA was 5 μM. Then, different concentrations of CdCl_2_ (0, 5, and 8 mg/mL) were added to the mixtures, and the mixtures were cultured at 20 °C. Subsequently, the fluorescent intensity of *C. elegans* was analyzed at 2 h and 6 h, respectively (excitation 490 nm and emission 525 nm).

### 2.7. Analysis of the Roles of ROS and NO in Cadmium Toxicity

IMZ and PY were used as ROS inhibitors to study the role of ROS in cadmium toxicity in *C. elegans*. *C. elegans* were exposed to 5 mg/mL of CdCl_2_ and incubated with 2.4 mM of IME and 10 mM of PY for 6 h at 20 °C. *C. elegans* were treated without CdCl_2_ or ROS inhibitor as the control. The vitality of *C. elegans* and the levels of ROS and NO in *C. elegans* were determined as described above.

L-NMMA is the inhibitor of NO synthase, and cPTIO is a NO scavenger [20]. L-NMMA and cPTIO were used to study the role of NO in Cd toxicity in *C. elegans*. *C. elegans* were exposed to 5 mg/mL of CdCl_2_ and incubated with 0.4 mM of L-NMMA and 0.2 mM of cPTIO for 6 h at 20 °C. *C. elegans* were treated without CdCl_2_, L-NMMA, or cPTIO as the control. Then, the vitality of *C. elegans* and the levels of ROS and NO in *C. elegans* were determined as described above.

### 2.8. The Intervention of V_C_ on Cadmium Toxicity

The protective role of V_C_ against CdCl_2_ in *C. elegans* was assessed. To observe these specific characteristics, pre-experiments were carried out. The V_C_ concentrations (0.5 and 1 mg/mL) were selected based on the results of the pre-experiments. *C. elegans* were exposed to 5 mg/mL of CdCl_2_ supplemented with V_C_ at various concentrations. The mixtures were incubated at 20 °C for 6 h, and *C. elegans* were treated without CdCl_2_ or V_C_ as the control. Finally, the vitality of *C. elegans* and the levels of ROS and NO in *C. elegans* were determined as described above.

### 2.9. The Intervention of Fruit Juices on Cadmium Toxicity

The protective role of fruit juices against CdCl_2_ in *C. elegans* was performed as follows. The concentrations of fruit juices were determined based on the results of the pre-experiments. *C. elegans* were exposed to 5 mg/mL of CdCl_2_ supplemented with fruit juices (50% (*v*/*v*) of the total volume of the medium). *C. elegans* were treated without CdCl_2_ or fruit juices as the control. The vitality of *C. elegans* and the levels of ROS and NO in *C. elegans* were determined as described above.

### 2.10. Statistical Analysis

Three biological replicates were performed for each experiment, and the results were presented as mean ± standard deviation. Significance of difference was analyzed by one-way analysis of variance and considered significant at *p* < 0.05.

## 3. Results

### 3.1. The Vitality of C. elegans Decreased Under CdCl_2_ Stress

**Figure 1:**
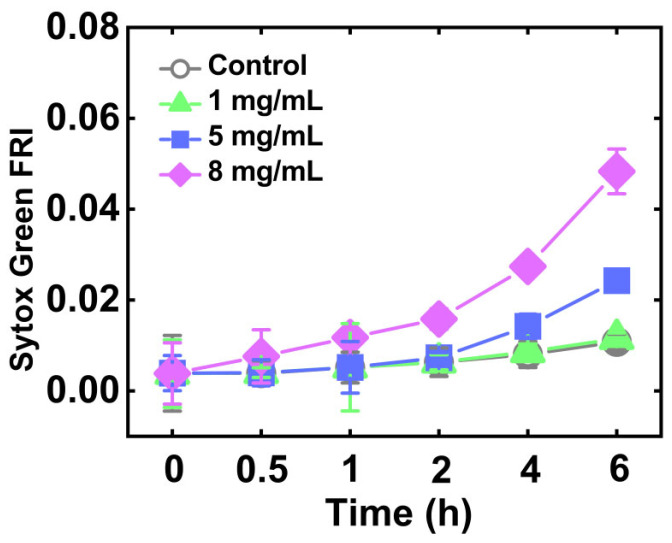
Vitality of *C. elegans* under the different concentrations of CdCl_2_. The stronger fluorescence intensity indicated the weaker vitality of nematodes.

SYTOX Green was used to study the vitality of *C. elegans* under Cd stress. The results (Figure 1) showed that the vitality of *C. elegans* decreased gradually with the increase of CdCl_2_ concentration and treatment time. There was no significant difference (*p* < 0.05) in fluorescence intensity between the group treated with 1 mg/mL of CdCl_2_ and the control group within 6 h, indicating that 1 mg/mL of CdCl_2_ had little effect on the vitality of *C. elegans*. The fluorescence intensity increased significantly after 6 h of treatment with 5 mg/mL of CdCl_2_ or 4 h of treatment with 8 mg/mL of CdCl_2_, indicating that a high concentration of CdCl_2_ would induce a decrease in the vitality of *C. elegans*.

### 3.2. The Accumulation of ROS and NO Under CdCl_2_ Stress

**Figure 2:**
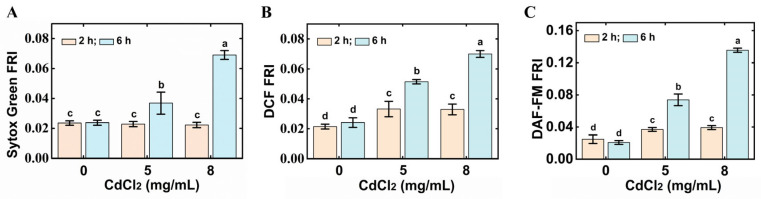
Effect of different concentrations of CdCl_2_ and different treatment times on *C. elegans*. (**A**) The vitality of *C. elegans* under CdCl_2_ stress. (**B**) The accumulation of ROS in *C. elegans* under CdCl_2_ stress. (**C**) The accumulation of NO in *C. elegans* under CdCl_2_ stress. The different letters indicate significant differences (*p* < 0.05).

Cd stress usually leads to intracellular oxidative stress, which will promote cell death [21]. To determine whether CdCl_2_ induces oxidative stress in nematodes, ROS and NO were detected by DCFH-DA and DAF-FM-DA, respectively. *C. elegans* were treated with different concentrations (5 mg/mL and 8 mg/mL) of CdCl_2_ for different times (2 h and 6 h); then, the intensities of fluorescence were detected, respectively. The results are shown in Figure 2. The vitality of *C. elegans* was inhibited with the increase of CdCl_2_ treatment times at concentrations of 8 mg/mL and had no significant difference at the concentrations of 5 mg/mL (Figure 2A). At the same time, with the increase of CdCl_2_ concentration, the accumulations of ROS and NO were increased (Figure 2B,C). The accumulation of ROS in nematodes increased significantly after being exposed to CdCl_2_ for 2 h, and nematodes showed a fluorescence intensity about 3.5-fold higher than the control after being exposed to 8 mg/mL of CdCl_2_ for 6 h. The accumulation of NO in nematodes did not significantly increase after being exposed to CdCl_2_ for 2 h. In comparison, the accumulation of NO in nematodes increased sharply after being exposed to CdCl_2_ for 6 h, and the fluorescence intensity was about 6.5-fold higher than that of the control group. The changing trend followed the change in the vitality of *C. elegans* under CdCl_2_ stress.

### 3.3. Reducing ROS and NO Levels in C. elegans Alleviates CdCl_2_ Toxicity

**Figure 3:**
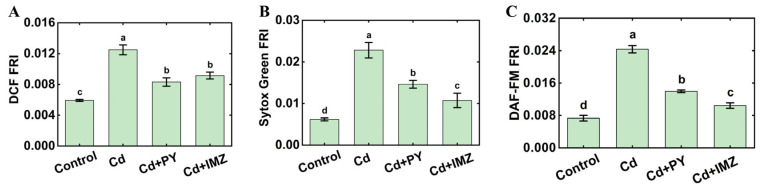
Effect of PY and IMZ on *C. elegans* under CdCl_2_ stress. (**A**) Effect of PY and IMZ on ROS accumulation in *C. elegans* under CdCl_2_ stress. (**B**) Vitality of *C. elegans* exposed to CdCl_2_ with the removal of ROS. (**C**) Effects of removal of ROS on NO production in *C. elegans* under CdCl_2_ stress. Control: *C. elegans* were treated with distilled water for 6 h; Cd: *C. elegans* were treated with 5 mg/mL of CdCl_2_ for 6 h; Cd + PY: *C. elegans* were treated with 5 mg/mL of CdCl_2_ and 10 mM of PY for 6 h; Cd + IMZ: *C. elegans* were treated with 5 mg/mL of CdCl_2_ and 2.4 mM of IMZ for 6 h. The different letters indicate significant differences (*p* < 0.05).

To detect the role of ROS in the toxicity of CdCl_2_ to nematodes, IMZ and PY were used as ROS inhibitors [22]. As shown in Figure 3A, IMZ and PY could significantly reduce the fluorescence of DCF in *C. elegans*, which means that the accumulation of ROS was reduced. The results also indicated that 10 mM of PY and 2.4 mM of IMZ could alleviate the vitality stress (Figure 3B).

**Figure 4:**
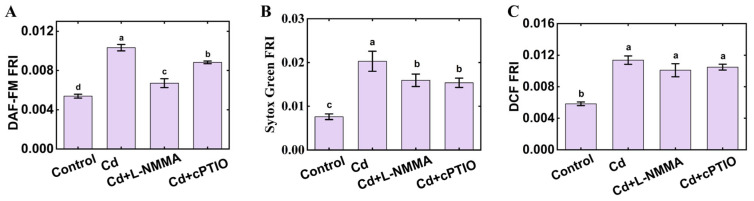
Effect of L-NMMA and cPTIO on *C. elegans* under CdCl_2_ stress. (**A**) Effects of L-NMMA and cPTIO on the accumulation of NO in *C. elegans* under CdCl_2_ stress. (**B**) Effects of L-NMMA and cPTIO on SYTOX Green fluorescence intensity in *C. elegans* under CdCl_2_ stress. (**C**) Effects of L-NMMA and cPTIO on the accumulation of ROS under CdCl_2_ stress. Control: *C. elegans* were treated with distilled water for 6 h; Cd: *C. elegans* were treated with 5 mg/mL of CdCl_2_ for 6 h; Cd + L-NMMA: *C. elegans* were treated with 5 mg/mL of CdCl_2_ and 0.4 mM of L-NMMA for 6 h; Cd + cPTIO: *C. elegans* were treated with 5 mg/mL of CdCl_2_ and 0.2 mM of cPTIO for 6 h. The different letters indicate significant differences (*p* < 0.05).

To further ascertain the role of NO under CdCl_2_ stress, the inhibitor and scavenger of NO were used. The accumulation of NO in *C. elegans* under CdCl_2_ stress was inhibited by L-NMMA and cPTIO (Figure 4A). At the same time, L-NMMA and cPTIO could moderate the vitality stress in *C. elegans* under CdCl_2_ stress (Figure 4B). These data suggested that the mechanism underlying acute CdCl_2_ poisoning in *C. elegans* was related to the accumulation of ROS and NO.

### 3.4. ROS Accumulation Drives NO Build-Up and Compromises Nematode Vitality

To clarify how ROS and NO interact during acute CdCl_2_ toxicity in *C. elegans*, we examined whether suppressing ROS affects NO levels and whether blocking NO influences ROS accumulation. We found that treatment with 10 mM PY or 2.4 mM IMZ, two ROS inhibitors reduced ROS accumulation, concurrently suppressed NO levels, and alleviated CdCl_2_-induced vitality loss in *C. elegans* (Figure 3). L-NMMA, as a NO inhibitor, and cPTIO, as a NO scavenger, attenuated both NO accumulation and the associated vitality decline, yet left the ROS levels unchanged (Figure 4). According to the above results, ROS plays a vital role in CdCl_2_ toxicity to *C. elegans*. We infer that CdCl_2_ could cause the accumulation of ROS in nematodes, further induce the outbreak of NO, and, finally, lead to a decrease in nematode activity and the death of nematodes.

### 3.5. Vitamin C and Fruit Juices Alleviate CdCl_2_ Poisoning

**Figure 5:**
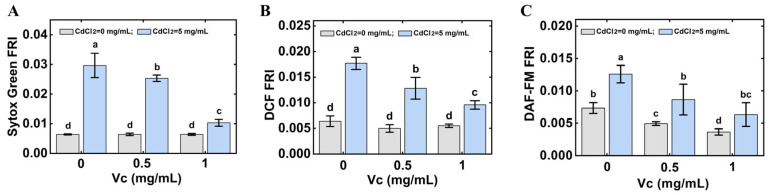
Effect of V_C_ on CdCl_2_ poisoning in *C. elegans*. (**A**) Effect of V_C_ on the vitality of *C. elegans* under CdCl_2_ stress. (**B**) Effect of V_C_ on the accumulation of ROS in *C. elegans* under CdCl_2_ stress. (**C**) Effect of V_C_ on the accumulation of NO in *C. elegans* under Cd stress. The different letters indicate significant differences (*p* < 0.05).

To evaluate the effect of V_C_ on CdCl_2_ poisoning, *C. elegans* were treated with 5 mg/mL of CdCl_2_ and different concentrations of V_C_ for 6 h. As shown in Figure 5A, *C. elegans* treated with CdCl_2_ and V_C_ showed a weaker fluorescence intensity of SYTOX Green than *C. elegans* under Cd stress, indicating that V_C_ could alleviate Cd poisoning in *C. elegans*, and the vitality of *C. elegans* under Cd stress increased gradually with the increase of V_C_ concentration. The simultaneous quantification of ROS and NO revealed that V_C_ markedly suppresses their accumulation in *C. elegans* under Cd stress (Figure 5B,C). These findings indicate that V_C_ confers Cd tolerance by attenuating ROS and NO accumulation, revealing a key protective mechanism.

**Figure 6:**
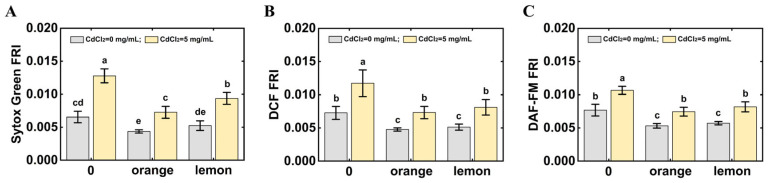
Effect of fruit juices on CdCl_2_ poisoning in *C. elegans*. (**A**) Effect of fruit juices on the vitality of *C. elegans* under CdCl_2_ stress. (**B**) Effect of fruit juices on the accumulation of ROS in *C. elegans* under CdCl_2_ stress. (**C**) Effect of fruit juices on the accumulation of NO in *C. elegans* under CdCl_2_ stress. The different letters indicate significant differences (*p* < 0.05).

The protective role of fruit juices against Cd in *C. elegans* was performed. The results are shown in Figure 6. It is determined that *C. elegans* treated with CdCl_2_ and juices showed higher vitality than *C. elegans* under CdCl_2_ stress, indicating that orange juice and lemon juice could ameliorate CdCl_2_ toxicity in *C. elegans*. The V_C_ content in orange juice was determined to be 52.36 mg/100 mL, whereas that in lemon juice was 51.55 mg/100 mL. Orange juice and lemon juice could also reduce the accumulation of ROS and NO in *C. elegans* under CdCl_2_ stress. Our results supported the hypothesis that V_C_ and fruit juices could alleviate CdCl_2_ poisoning.

## 4. Discussion

Cd pollution, one of the most critical environmental problems, poses a threat to food safety and human health. Microbial toxicity assays constitute an indispensable pillar within the environmental risk-assessment framework for chemical contaminants [23]. Compared with microorganisms, C. *elegans* exhibit many responses similar to those observed in vertebrate systems. *C. elegans*, a model organism, has been widely used to analyze the harm of the heavy metal [24].

In this study, *C. elegans* was used as an in vivo model. We delineated a sequential toxicity pathway for CdCl_2_: CdCl_2_ exposure → ROS burst → NO generation → vitality decline. ROS was identified as the primary trigger, whereas NO acted as a downstream amplifier. Both V_C_ and V_C_-rich natural juices effectively scavenged ROS and NO, fully restoring the vitality of *C. elegans*. These results provide direct evidence that dietary antioxidants can antagonize acute Cd toxicity.

The inhibition of ROS simultaneously reduced NO levels and rescued vitality, whereas NO scavengers improved vitality without altering ROS, confirming the upstream position of ROS. In general, heavy metals could lead to oxidative damage, and the damages are mediated by the production of ROS [25,26]. It has been reported that Cd could deplete glutathione and protein-bound sulfhydryl groups, resulting in the production of ROS [25]. Free radicals play several beneficial roles for the organism when they are maintained at low or moderate concentrations. However, when the concentration is higher than the normal state, oxidative stress will induce a variety of diseases, accelerating the process of body aging and leading to acute allergic reactions [27]. The production of ROS could also result in enhanced lipid peroxidation, DNA damage, and the disruption of sulfhydryl homeostasis. Subsequent investigations are warranted to track temporal variations in these indicators quantitatively.

It was discovered that *C. elegans* under CdCl_2_ stress were also accompanied by the accumulation of NO, and the level of NO was also related to the vitality of nematodes. Several studies indicate that endogenous NO can contribute to Cd toxicity in plants [28,29]. The endogenous NO has been considered a signaling molecule triggering cell death in mammals. Therefore, it is attractive to study the level of NO in animal models exposed to Cd. Other than amplifying oxidative damage, NO can form peroxynitrite (ONOO^−^) with O_2_•^−^. Future work should employ ONOO^−^-specific fluorescent probes to quantify the production of ONOO^−^.

V_C_ shows excellent potential to protect against Cd-induced damage in rats [30]. V_C_-rich fruit juices may pose detoxifying potential. Our study provides compelling evidence that both V_C_ and V_C_-rich fruit juices restore *C. elegans* vitality and markedly suppress CdCl_2_-elicited ROS and NO accumulation. V_C_ is a nutritional supplement, yet excessive intake can be toxic. The acute toxic dose low (TDLo) in humans is 900 mg/kg (https://pubchem.ncbi.nlm.nih.gov/compound/54670067#section=Adverse-Effects, accessed on 16 August 2025). The highest concentration used in this study to antagonize acute Cd toxicity was only 1 mg/mL, which is far below that limit. ADI of V_C_ is not specified, as intake from food is not considered a health hazard (https://pubchem.ncbi.nlm.nih.gov/compound/54670067#section=Exposure-Control-and-Personal-Protection, accessed on 16 August 2025). Oranges and lemons are characterized by a rich array of natural compounds, including ascorbic acid, citric acid, polyphenols, essential oils, and notably abundant flavonoids [31]. It has been reported that citric acid exerts a protective effect against oxidative damage triggered by heavy metal exposure in *C*. *elegans* [32], and polyphenols exhibit remarkable antioxidant activity. An amount of 0.5 mg/mL V_C_ significantly restored vitality, and juices applied at half volume delivered about 0.26 mg/mL V_C_ yet performed comparably. The results suggest that V_C_ synergizes with natural compounds such as citric acid and polyphenols. This synergistic effect points to a critical direction for further investigation.

This study examined cadmium toxicity induced by high-dose exposure and employed a single animal model. It lacks validation in mammals or at the cellular level. Because *C. elegans* possesses neither livers nor kidneys, the primary organs for cadmium accumulation, which are toxicokinetic, differ markedly from those in humans. The mechanistic analysis relied solely on ROS/NO fluorescent probes and ROS/NO inhibitors, without a systematic dissection of downstream signaling pathways. Therefore, future research should focus on exploring the detailed molecular pathways involved in Cd-induced ROS and NO production, as well as investigating the long-term protective effects of V_C_ and fruit juices in more complex biological models. Specifically, transgenic lines can be employed to determine whether V_C_ exerts its protective effects via defined signaling cascades. At the same time, RNA-seq-based profiling of differentially expressed genes can further validate the ROS/NO molecular network. Moreover, the investigation should be extended to higher animal models, such as mice, to compare the cadmium-toxicity-mitigating efficacy of V_C_ and fruit juices, as well as V_C_-deficient diets, thereby providing robust evidence for population-level nutritional interventions.

## 5. Conclusions

This study provides comprehensive insights into the mechanisms underlying Cd-induced toxicity in *C. elegans* and explores the protective effects of V_C_ and fruit juices against Cd-induced oxidative stress and toxicity. Our results demonstrate that Cd exposure (5 mg/mL and 8 mg/mL of CdCl_2_) significantly impairs the vitality of *C. elegans* within a short period (6 h), primarily through the accumulation of ROS and NO. The findings that the inhibition of ROS not only reduces NO levels but also restores the vitality of *C. elegans*—whereas the removal of NO only improves vitality without affecting ROS levels—strongly suggest that ROS accumulation is the primary event in Cd-induced toxicity, while NO acts as a secondary contributor to the overall toxic response. Moreover, our study highlights the potential of V_C_ and V_C_-rich fruit juices as effective interventions against Cd-induced toxicity. Both V_C_ and fruit juices significantly enhance the vitality of *C. elegans* under CdCl_2_ stress by effectively scavenging ROS and NO. This indicates that the antioxidant properties of V_C_-rich materials play a crucial role in mitigating Cd-induced oxidative stress and toxicity.

## Data Availability

The original contributions presented in the study are included in the article, further inquiries can be directed to the corresponding authors.
